# Preventing Neurodegeneration by Controlling Oxidative Stress: The Role of OXR1

**DOI:** 10.3389/fnins.2020.611904

**Published:** 2020-12-15

**Authors:** Michael R. Volkert, David J. Crowley

**Affiliations:** ^1^Department of Microbiology and Physiological Systems, University of Massachusetts Medical School, Worcester, MA, United States; ^2^Department of Biological and Physical Sciences, Assumption University, Worcester, MA, United States

**Keywords:** gene therapy, *OXR1*, oxidative stress resistance, reactive oxygen species, neurodegeneration, retinal degeneration

## Abstract

Parkinson’s disease, diabetic retinopathy, hyperoxia induced retinopathy, and neuronal damage resulting from ischemia are among the notable neurodegenerative diseases in which oxidative stress occurs shortly before the onset of neurodegeneration. A shared feature of these diseases is the depletion of *OXR1* (oxidation resistance 1) gene products shortly before the onset of neurodegeneration. In animal models of these diseases, restoration of *OXR1* has been shown to reduce or eliminate the deleterious effects of oxidative stress induced cell death, delay the onset of symptoms, and reduce overall severity. Moreover, increasing *OXR1* expression in cells further increases oxidative stress resistance and delays onset of disease while showing no detectable side effects. Thus, restoring or increasing *OXR1* function shows promise as a therapeutic for multiple neurodegenerative diseases. This review examines the role of *OXR1* in oxidative stress resistance and its impact on neurodegenerative diseases. We describe the potential of OXR1 as a therapeutic in light of our current understanding of its function at the cellular and molecular level and propose a possible cascade of molecular events linked to *OXR1*’s regulatory functions.

## Introduction

*OXR1* is emerging as a key player in the pathology of neurodegenerative diseases. It was discovered as a gene required for oxidative stress resistance in eukaryotes 20 years ago (Volkert et al., 2000), but interest in *OXR1* has expanded as its role in neurodegeneration has become more widely recognized. Most recent discoveries of the key roles of *OXR1* began as investigations into the genetic mechanisms behind specific neurodegenerative diseases. These studies led to the conclusion that *OXR1* prevents progression of neurodegeneration by reducing oxidative stress and the damage and cell death that oxidative stress causes. In many of the diseases in which *OXR1* plays a role, its mRNA and protein levels decline shortly before the initiation of oxidative stress and neurodegeneration. Restoration of *OXR1* expression prevents or delays the onset of the pathology (Murray et al., 2013; Puspita et al., 2017; Mo et al., 2019).

*OXR1* has been implicated in a growing list of neurodegenerative diseases such as oxygen-induced retinopathy, diabetic retinopathy, Parkinson’s disease, ischemia-induced neuronal damage, and amyotrophic lateral sclerosis (ALS) (Natoli et al., 2008a; Murray et al., 2013; Finelli et al., 2015; Liu et al., 2015; Puspita et al., 2017; Jiang et al., 2019; Williamson et al., 2019). *OXR1*’s ability to reduce oxidative stress and neurodegeneration in multiple diseases strongly suggests that it can be an effective therapeutic target. Increasing *OXR1* expression has also been shown to delay Lupus-associated induced kidney failure, which is associated with oxidative stress (Li et al., 2014), suggesting *OXR1* may have therapeutic applications beyond treating neurodegenerative diseases.

The role of *OXR1* in these diseases is multifaceted. One of its major functions is to control the expression of genes that alleviate oxidative stress by increasing cellular resistance to reactive oxygen species (ROS) and the stress these molecules cause the cell. Oxidative stress occurs when levels of ROS, including hydroxyl radicals, superoxide, singlet oxygen, and other reactive forms of oxygen, exceed the cell’s capacity to detoxify and inactivate these molecules and the cell’s ability to repair the damage they cause to lipids, proteins, and nucleic acids. Oxidative stress has long been implicated as a factor that contributes to the pathology of many neurodegenerative diseases including Alzheimer’s disease, Parkinson’s disease, ALS, ‘Huntington’s disease, Down’s syndrome, and multiple sclerosis (Butterfield, 2006; Melo et al., 2011; Butterfield et al., 2012; Kim et al., 2015; Ohl et al., 2016). Whether *OXR1* plays a role in all of these neurodegenerative diseases remains to be determined.

### Discovery and Demonstration That *OXR1* Is Required for Oxidative Stress Resistance

*OXR1* was discovered in a search for human genes that could prevent or repair spontaneous oxidative damage in an *E. coli* strain engineered to report the level of oxidative mutagenesis (Volkert et al., 2000, 2008; Elliott and Volkert, 2004). Further studies of the yeast *OXR1* homolog confirmed its requirement for oxidative stress resistance in eukaryotes (Elliott and Volkert, 2004). The ability of the human homolog to restore oxidative stress resistance to a yeast *oxr1* deletion strain suggested its function in stress resistance is conserved among the eukaryotic OXR1 proteins (Elliott and Volkert, 2004). This conservation of function has been substantiated in a number of studies of invertebrates including mosquitos, *Drosophila*, silkworms, and *C. elegans*, as well as the non-mammalian vertebrate zebrafish (Jaramillo-Gutierrez et al., 2010; Wang et al., 2012, 2019; Kobayashi et al., 2014; Sanada et al., 2014; Su et al., 2017; Xu et al., 2020). A role for *OXR1* in regulation of oxidative stress response genes has been demonstrated in the mosquito, fruit fly, zebrafish, and mammals (Jaramillo-Gutierrez et al., 2010; Wang et al., 2012, 2019; Kobayashi et al., 2014; Yang et al., 2014, 2015; Matsui et al., 2020; Xu et al., 2020). Thus, the basic functions of OXR1 in oxidative stress resistance are highly conserved in eukaryotes.

#### *OXR1* Gene Structure

**FIGURE 1:**
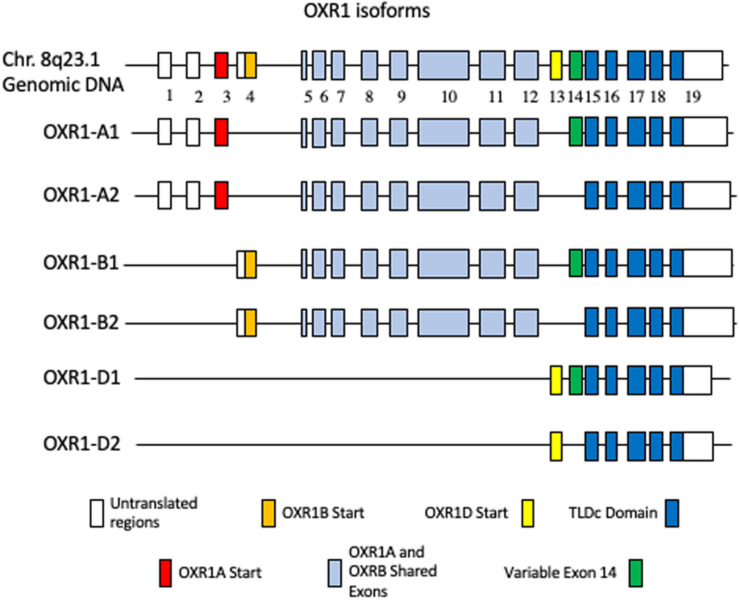
Human OXR1 Isoforms. White boxes depict non-coding exons or exon regions. The first coding exons of: OXR1A1 (Accession no. NP_001185462.1) and OXR1A2 (Accession no. NP_060472.2) (red); OXR1B1 (Accession no. XP_016869078.1) and OXR1B2 (Accession no. NP_851999.2) (gold); OXR1D1 (Accession no. NP_001185463) and OXR1D2 (Accession no. NP_001185464.1) (yellow); variable exon 14 (green); TLDc Domain (dark blue); exons shared by A and B forms (light blue) are highlighted.

Like many mammalian genes, human *OXR1* expresses multiple isoforms. Figure 1 shows the long (A and B) and short (D) forms of human *OXR1* (Elliott and Volkert, 2004; Oliver et al., 2011; Yang et al., 2014). An additional human *OXR1*C form has been found only in testes and has not been studied extensively to date (Yang et al., 2014). These isoforms correspond to the mRNA species identified in humans to date. The A forms appear to be primarily brain specific forms (Elliott and Volkert, 2004; Yang et al., 2014). In this review we have designated them A1 and A2, which differ only by the presence of the 81 bp exon 14 (A1), or its absence (A2). The A1 and A2 forms encode proteins of 873 and 846 amino acids. The B forms are more widely expressed and are typically found in cells of tissues that have high levels of oxidative metabolic activity (Elliott and Volkert, 2004; Yang et al., 2014; Finelli et al., 2016). As is the case with the A forms, B1 and B2 differ by the presence or absence of exon 14 and produce proteins of 866 and 839 amino acids, respectively.

The short D forms are present in multiple tissues and appear to be expressed from their own promoter (Finelli et al., 2015). They begin with exon 13, which is not found in longer forms of *OXR1*. The *OXR1D* forms (called *Oxr1C* in mouse) are also produced with and without exon 14 (*OXR1D1* and *OXR1D2*, respectively) and produce proteins of 243 and 216 amino acids. All of the human A, B, C, and D isoforms contain the C-terminal TLDc domain, a highly conserved domain of unknown function (Durand et al., 2007; Finelli and Oliver, 2017). A recent review describes and compares the complete family of proteins containing TLDc domains and their roles in neurodegenerative diseases in detail (Finelli and Oliver, 2017).

Studies of *OXR1* often have not clearly distinguished the specific isoforms, instead discriminating primarily between the long and short forms. The A and B forms are typically described as the long forms and the D forms (and the corresponding mouse *Oxr1*C forms) are frequently called the short forms. Therefore, potential differences beyond tissue specificity between the A, B, C, and D forms of *OXR1*, or the roles of exons 13 and 14 are not presently known. Although the mRNA of the A and B forms are sufficiently different in length to allow their discrimination on northern blots, the proteins produced from these mRNAs are very similar in size making them difficult to distinguish by Western blot analysis. Western blots using antibodies specific for the OXR1 TLDc domain typically reveal proteins in mice that are approximately 85, 55, 40, and 24 kDa (Oliver et al., 2011). While the largest and the smallest forms are thought to correspond to the OXR1B and D isoforms, the intermediates have not been characterized to date. Whether these are truncated proteins, post-translationally processed forms, or additional mouse-specific splice variants is not clear.

#### OXR1 Homologs

A number of the TLDc family proteins are highly expressed in neurons and many have been associated with neurological defects and/or diseases (Finelli et al., 2016; Finelli and Oliver, 2017). The importance of the TLDc domain was demonstrated in *Drosophila* by Wang et al. (2012), who found that the phenotype resulting from deletion of the *Drosophila OXR1* homolog *mtd* could be complemented by expression of isoforms containing essentially just the TLDc domain (Wang et al., 2012, 2019). Similarly, expression of either the long or short forms of *Oxr1* prevents neurodegeneration in Oxr1-deleted *Bella* mouse (Oliver et al., 2011; Finelli et al., 2016). To further test the importance of the TLDc domain, Finelli et al. (2016) produced an insertion mutation in mice that deleted the TLDc domain, which inactivated *OXR1’*s neuroprotective functions. These results demonstrate that the TLDc domain plays a critical role for OXR1 to function in neuroprotection, however the mechanism of action remains elusive (for review see Finelli and Oliver, 2017).

In this review, we focus primarily on the *OXR1* gene and its closest homolog, the nuclear coactivator 7 (NCOA7). Alignment of the NCOA7 and OXR1B2 proteins reveals that 11 of the 16 exons are at least 40–70% identical in sequence (Durand et al., 2007). The *NCOA7* genomic structure also has a similar pattern of size and spacing of exons and introns as *OXR1* and produces structurally similar long and short forms, suggesting that *OXR1* and *NCOA7* likely arose from gene duplication (Durand et al., 2007; Shkolnik et al., 2008; Yu et al., 2014). The short form of *NCOA7* begins with a unique exon spliced to the TLDc domain (Shkolnik et al., 2008; Yu et al., 2014). However, the amino acid sequences of the unique first exons of the short forms of OXR1 and NCOA7 proteins do not share any homology. The short form of *NCOA7* is transcribed from its own promoter, which is predicted to be the case for *OXR1D* as well (Yu et al., 2014; Finelli et al., 2015). Like *OXR1*, *NCOA7* is highly expressed in most brain neurons (Shao et al., 2002). Both OXR1 and NCOA7 contain the highly conserved C-terminal TLDc domain.

#### *OXR1* Induction

*OXR1* is induced by oxidative stress, but the mechanism that regulates this induction remains unclear. Increasing OXR1 levels by the introduction of an additional copy of *OXR1* transcribed from a strong promoter increases cellular resistance to oxidative stress, suggesting elevated OXR1 levels are critical for this function (Finelli et al., 2016). In N2a mouse neuroblastoma cells in culture, both the long and short forms of *OXR1* are induced by oxidative stress treatment, however, the timing of induction of these isoforms is different. The long forms are induced within 30 min after treatment with an oxidative agent, whereas the short forms are induced only after 2 h, suggesting they may be independently regulated, respond to different levels of oxidative stress, or are required at different stages of stress mitigation (Finelli et al., 2015). Neither the short nor the long forms of *NCOA7* are induced by hydrogen peroxide (Durand et al., 2007; Yu et al., 2014), but the long form is induced by arsenite treatment (Finelli et al., 2016), suggesting that the *NCOA7* response to oxidative stress differs from that of *OXR1*, which responds to treatment by both oxidative agents.

The importance of elevated levels of *OXR1* to disease outcome was demonstrated in a study by Liu et al. of the SOD1^G93A^ mouse model of ALS (Liu et al., 2015). This mouse, like humans with ALS (Oliver et al., 2011), expresses OXR1 at elevated levels, suggesting they have induced the OXR1 response and are undergoing oxidative stress. Liu et al. constructed and tested a mouse that carries both the SOD1^G93A^ allele and a transgene expressing *Oxr1* from a strong neuron-specific promoter. In this mouse the onset of disease was delayed, and its lifespan was increased. This suggests that elevating OXR1 protein to levels higher than the those normally attained when the endogenous *OXR1* gene is induced provides further protection from neurodegeneration (Liu et al., 2015). In humans the importance of the level of OXR1 is underscored by the recent study by Chen et al. in which patients carrying a heterozygous *OXR1* mutation that prevents expression of one copy of the OXR1D isoform were found to have an impairment in the development of language skills, suggesting that haploinsufficiency of only this isoform results in a detectable neurological impairment which is less severe when compared to the phenotype of patients that lack expression of all OXR1 isoforms (Chen et al., 2017; Wang et al., 2019). Therefore, a key to understanding *OXR1* function is to determine how *OXR1* expression is regulated. Are there upstream regulatory factors that are required, or does *OXR1* function in an autoregulatory pathway in response to oxidative stress?

## *OXR1* Is Required for Neuron Viability

### *OXR1* Is Required for Granular Cell Layer Neuron Viability in the Cerebellum

The high-level expression of *OXR1* in the brain, specifically in neurons together with its role in reducing oxidative stress sensitivity, led to the suggestion that *OXR1* may play a role in mitigating oxidative stress-induced neurodegeneration (Elliott and Volkert, 2004; Durand et al., 2007; Oliver et al., 2011). The role of *OXR1* in neurodegeneration was firmly established by the discovery of the *Bella* mouse. Oliver et al. (2011) identified this mouse model from a screen for movement disorders and ataxia in mutagenized mice. The *Bella* mouse exhibited a rapidly progressing ataxia that began at approximately 2 weeks of age, resulting in severe ataxia and death by 4 weeks of age. Although the mouse carried a deletion of two genes including *Oxr1*, reintroduction of *Oxr1* complemented the phenotype and reversed the effects of the deletion, restoring the *Bella* mouse to health and demonstrating that the pathology was due to loss of *Oxr1* (Oliver et al., 2011). Construction of a mouse in which the C-terminal region of *Oxr1* was deleted resulted in a phenotype identical to that of the *Bella* mouse and confirmed the role of *Oxr1* and the importance of the TLDc domain in reducing oxidative stress and preventing neurodegeneration (Finelli et al., 2016).

The ataxia of the *Bella* mouse appears to result from oxidative stress-induced degeneration of the granule cell layer (GCL) neurons of the cerebellum that are involved in motor control (Hashimoto et al., 1999; Ohgoh et al., 2000; Oliver et al., 2011; Yamashita et al., 2011; Bodranghien et al., 2016). The time of onset of the phenotype coincides with the approximate time that these neurons are thought to stop dividing in mice, complete their migration to their final location, and become mature, terminally differentiated neurons (Ding et al., 2013; Leto et al., 2016). It is at this time that the GCL neurons accumulate oxidative damage and begin to die possibly because of their high levels of oxidative metabolism and low levels of antioxidant enzymes (for review see DiMauro and Schon, 2008; Watts et al., 2018).

It is unclear why only the cerebellar GCL neurons are particularly vulnerable to oxidative stress and neurodegeneration since most brain neurons express *OXR1* at high levels. The early death of the *Bella* mouse may prevent detection of damage to other neurons possibly because they have lower levels of ROS or higher levels of other protective proteins compensating for the loss of OXR1. One possible explanation for the sensitivity of the GCL neurons comes from the observation that their NCOA7 protein levels are relatively low compared to other brain neurons (Protein Atlas^1^; Finelli et al., 2016). The high degree of homology between *NCOA7* and *OXR1* raises the possibility that NCOA7 may compensate, at least to some extent, for the loss of OXR1 function by providing some protection from oxidative stress in neurons where it is more highly expressed than in GCL neurons. The ability of *NCOA7* to complement *OXR1* is further supported by the results of Wang et al. (2019). They tested the ability of human *OXR1* and *NCOA7* to complement the phenotype of *Drosophila* carrying a deletion of the mustard (*mtd*) gene, the fly homolog of OXR1. Most *mtd* mutant flies die at or before the eclosion stage (emergence from the pupae). The few flies that do emerge from their pupae die shortly thereafter. The short forms of human OXR1 and NCOA7 were capable of restoring viability to the *mtd* mutant flies demonstrating that only the TLDc domain was necessary. This further supports the notion that these two human genes have overlapping functions. This needs to be tested by simultaneously modulating *NCOA7* and *OXR1* expression in mammalian cells and testing oxidative stress resistance levels.

### *OXR1* Is Required for Retinal Neuron Viability

In addition to its role in protection of brain neurons, *OXR1* appears to play a key role in several neurodegenerative diseases of the retina. The first study demonstrating a role for *OXR1* in retinal degeneration used C57bl6/J mice. These mice develop blindness when raised in high (75%) oxygen levels and have been used as models for retinopathy of prematurity (Natoli et al., 2008a,b), which can occur in preterm infants that require supplemental oxygen for survival (for review see Hellstrom et al., 2013). C57bl6/J sensitive mice and the Balb/c resistant control mice used in this study induce *OXR1* mRNA and protein to high levels upon exposure to high oxygen and their *OXR1* mRNA and protein levels remain high during the period when the photoreceptors are resistant to hyperoxia. However, by approximately 14 days, *OXR1* mRNA and protein levels are reduced in the C57bl6/J mutant mouse to levels below those measured prior to oxygen exposure and retinal degeneration ensues. In the Balb/c control mice, OXR1 levels remain high and no retinal changes are detectable. While this observation was primarily correlational, it does suggest that *OXR1* levels play a role in the protection of photoreceptors from hyperoxia-induced death (Natoli et al., 2008a).

A subsequent study of diabetic retinopathy more clearly demonstrates a causal effect of *OXR1* in retinal degeneration. Murray et al. (2013) studied microRNA expression in the Akita mouse model of diabetic retinopathy and found that the microRNA miR-200b was induced shortly before the onset of retinopathy. miR-200b destabilized *OXR1* mRNA and reduced expression of the protein. As the *OXR1* mRNA and protein levels declined, the retinal neurons accumulated an excess of oxidative DNA damage resulting in the initiation of apoptosis and retinal neuron death. Like most microRNAs, miR-200b destabilizes its target mRNAs resulting in reduced levels of multiple mRNAs and proteins. To determine if *OXR1* was a key factor leading to the oxidative damage and apoptosis, Murray et al. mimicked the phenotype by introducing miR-200b to cultured retinal Muller cells. This caused a rapid accumulation of oxidative damage and increased apoptosis similar to that seen in the retina of the Akita mouse. Introducing a miR-200b resistant form of *OXR1* along with the miR-200b to the Muller cells caused a reduction the level of oxidative damage and apoptosis. This demonstrates that *OXR1* expression confers resistance and enhances viability and that its depletion is a major cause of cell death.

### Depletion of *OXR1* Affects Neurodegeneration, and Cell Death

Two studies examined gene expression in several different brain regions of post-mortem samples from Parkinson’s patients and compared these with age matched controls. In all cell types *OXR1* mRNA levels were lower in the Parkinson’s groups compared to matched controls (Zhang et al., 2005; Stamper et al., 2008). These were most significant in the Broadman’s area 9 neurons where OXR1 was 5.3-fold lower in the Parkinson’s patients and in the posterior cingulate cortex pyramidal neurons where the Parkinson’s patients without dementia exhibited a 3.0-fold reduction in *OXR1* mRNA and those with dementia showed a 2.0-fold reduction. Other brain regions showed minor reductions in OXR1 levels of 1.7-fold (substantia nigra) and 1.4-fold (putamen).

The mechanism of *OXR1* reduction in Parkinson’s was studied by Li et al. (2017). They demonstrated that an increased level of the *OXR1*-targeting microRNA miR-137 is found in exosomes in serum of Parkinson’s patients compared to controls. Their subsequent study demonstrated that exosomes can transport miR-137 into brain neurons (Jiang et al., 2019). Exosomes containing miR-137 are also found in the bloodstream of mice treated with methyl-4-phenyl-1,2,3,6-tetrahydropyridine (MPTP), a chemical that produces a Parkinson’s-like disease in these animals as well as in humans (for review see Langston, 2017). The MPTP-treated mice had higher levels of oxidative damage and apoptosis in their brain tissue along with a loss of Nissl bodies in the neurons of the substantia nigra. MPTP-treated mice showed increased tremors, decreased pole climbing ability and decreased traction time when compared to control mice. These MPTP-induced cellular changes and Parkinson’s-like symptoms could be reversed by injection of a lentivirus that over expressed *OXR1* into the brain, strongly suggesting that the pathological effects of miR-137 are due to its inhibition of *OXR1* (Jiang et al., 2019).

A study by Mo et al. (2019) demonstrated that *OXR1* repression increases the pathological consequences of ischemia in a rat model. For reasons that are unclear, ischemia causes the induction of the microRNA miR-365 which, like miR-137 and miR-200b, down regulates *OXR1*. The key experiments demonstrated that miR-365 is induced upon occlusion of the middle cerebral artery and that this microRNA represses the expression of *OXR1*. Mo et al. then demonstrated that an antagomir that blocks the ability of miR-365 to repress gene expression restored *OXR1* levels and reduced the extent of the oxidative damage as measured in brain tissue by (γH2-AX levels, a marker of DNA damage. Measurement of the infarct zone showed that the extent of brain damage was also reduced. Mo et al. (2019) concluded that the effects of miR-365 were specifically due to repression of OXR1 expression by adding an OXR1 specific siRNA along with the antagomir. When OXR1 expression is repressed by the siRNA, the antagomir was no longer able to provide protection indicating that OXR1 expression is required to reduce brain damage resulting from ischemia.

Two additional studies provide further support for a key role for OXR1 in neurotoxicity and cell death. Sevoflurane is a common anesthetic that has been associated with neurotoxicity in rodents and humans (Bahmad et al., 2020; Sun, 2010). In human hippocampal cells in culture, sevoflurane induces miR-302e, yet another microRNA that represses OXR1 mRNA and protein levels. Sevoflurane treatment increases levels of oxidative damage and cell death. However, experimentally increasing *OXR1* levels abolished the ROS-associated toxicity of sevoflurane (Yang et al., 2018). Piperlongumine functions by a completely different mechanism. It is a compound that kills senescent cells because of their elevated permeability to this drug. Piperlongumine binds directly to the OXR1 protein, destabilizes it, and targets it for degradation. In all of the above disease and cell death models, once OXR1 protein levels decline, oxidative stress ensues, triggering apoptosis and cell death (Zhang et al., 2018).

### *OXR1* Is Critical for Oxidative Stress Resistance of Neurons

The above studies demonstrate that *OXR1* expression is critical for the survival of neurons experiencing oxidative stress. In fact, when *OXR1* is completely absent, as is the case in *OXR1*-deleted mice, the GCL neurons of the cerebellum cannot survive once they become mature neurons, possibly because of their high levels of oxidative metabolism and low levels of antioxidant enzymes (DiMauro and Schon, 2008; Oliver et al., 2011; Watts et al., 2018). A reduction in *OXR1* levels, either by elevated expression of a number of different microRNAs or by destabilization of the protein, increases cellular sensitivity to oxidative stress, apoptosis, and cell death. In animal models this leads to neurodegeneration.

In humans, mutations that cause bi-allelic loss of function and absence of *OXR1* expression result in a severe neurological phenotype that manifests itself as developmental delay, intellectual disability, language delay, cerebellar atrophy and seizures. This suggests that in humans *OXR1* plays a role during development that results in neurological defects (Wang et al., 2019). Human patients from a family that carried the same heterozygous early termination mutation in the unique first exon of OXR1D have been identified. These patients have language impairments, suggesting haploinsufficiency of this form of OXR1 leads to a neurological phenotype (Chen et al., 2017). Developmental defects have also been noted in zebrafish and *Drosophila OXR1* mutants (Wang et al., 2012, 2019; Xu et al., 2020). It is not clear if the developmental defects are due to role of *OXR1* in oxidative stress resistance, or if *OXR1* has additional functions required for normal neurological development.

To more fully understand the role of *OXR1* in neurodegenerative diseases much remains to be learned about how *OXR1* functions at the cellular and molecular level. The information that is available does lead to a speculative model of how *OXR1* may function to protect cells from oxidative stress induced death given the roles it plays in gene expression and function which will be discussed below.

## Molecular and Cellular Biology of *OXR1*

### How Does *OXR1* Function at the Molecular and Cellular Level?

Although the specific molecular actions of the *OXR1* isoforms are not completely understood, three very important and complementary mechanisms are beginning to emerge. These include its role in controlling expression of oxidative stress resistance genes by acting on the regulatory elements that are required for their induction or repression, the possibility that it acts as a cellular sensor of oxidative stress, and its protein-protein interactions that directly affect the function of OXR1’s interacting partner proteins.

### *OXR1*-Dependent Regulation of Gene Expression

A variety of studies show that *OXR1* has a regulatory role that is involved in the control of a number of stress response genes associated with survival during oxidative stress. A role for *OXR1* in the regulation of gene expression was first demonstrated in mosquitoes by Jaramillo-Gutierrez et al. (2010). A deletion of *OXR1* sensitized mosquitoes to H_2_O_2_ in their drinking water. This phenotype was associated with an inability to induce a set of genes, including glutathione peroxidase (GPX) and catalase, both required for oxidative stress resistance. A regulatory role was also suggested by the results of Oliver and coworkers who found that the failing GCL neurons of the *Bella* mouse had about a 70% reduction in the level of GPX1, an ROS-detoxifying enzyme (Oliver et al., 2011).

A regulatory function for *OXR1* in mammalian cells was directly demonstrated by two recent studies (Yang et al., 2014, 2015; Matsui et al., 2020). These studies showed that suppression of *OXR1* by an siRNA in HeLa cells caused sensitivity to oxidative stress, reduced expression of the cyclin-dependent kinase (*p21/CIP1/WAF1)*, *GPX2*, and heme oxygenase (*HO-1/HMOX1*), and abbreviated the G2/M checkpoint arrest, either after peroxide treatment, or gamma irradiation (Yang et al., 2014, 2015; Matsui et al., 2020). Yang et al. (2015) demonstrated that *OXR1* functions upstream of regulatory proteins such as HIF1A, SP6, E2F8, TCF3, p21, Fos, and Jun, that control a variety of stress responses. The nuclear factor erythroid 2-related factor 2 (*Nrf2*) is known to regulate a number of oxidative stress genes (for review see Tonelli et al., 2018; He et al., 2020), including *GPX2* and *HMOX1*. Both of these genes are no longer inducible when OXR1 is depleted, suggesting that OXR1 may also be required for the induction of Nrf2-dependent oxidative stress resistance gene expression (Bryan et al., 2013; Xiong et al., 2015). A role for OXR1 in Nrf2-dependent regulation is further supported by the observation that OXR1 interacts directly with the Keap1 protein, which regulates Nrf2 activity (Yang et al., 2020).

Among the genes that are differentially expressed in an OXR1-depleted HeLa cell line are those that metabolize or detoxify ROS, genes that affect apoptosis, genes involved in autophagy, and genes of the p53 response that control the cell cycle during oxidative stress. OXR1 enhances DNA repair, cell cycle arrest, ROS detoxification, and other resistance mechanisms, while repressing apoptosis (Yang et al., 2015). Thus, *OXR1* functions to activate a multifaceted response to oxidative stress, apparently by controlling multiple transcriptional regulators that in turn function to control the expression of genes that increase oxidative stress resistance and cell viability.

A recent study by Yang et al. (2020) provides further insights into OXR1’s regulatory mechanism. This study shows that in human U2OS osteosarcoma cells and rat GH3 pituitary tumor cells, OXR1 interacts with protein methyl transferase 5 (PRMT5) and stimulates its activity after peroxide treatment. PRMT5 methylates arginine residues within histones H3 and H4 to modulate chromatin structure, a key step in transcriptional activation of genes (Lorton and Shechter, 2019). Their conclusion that the OXR1A isoform is a coactivator of PRMT5 raises the possibility that OXR1 may affect gene expression by modulating histone methylation and chromatin structure. Both OXR1 and PRMT5 affect transcription both positively and negatively (Blanc and Richard, 2017; Yang et al., 2020). OXR1 was also shown to stimulate PRMT5-dependent methylation of p53, which results in cell cycle arrest. p53 also controls genes involved in DNA repair and apoptosis, increasing repair and repressing apoptosis (Berger, 2008; Jansson et al., 2008). This raises the possibility that the repression of the cell cycle and inhibition of apoptosis upon oxidative damage, which requires OXR1, may be mediated through its interaction with PRMT5 and p53 (Yang et al., 2015, 2020). OXR1 protein interacts with PRMT5 in both the cytoplasm and nucleus, potentially implicating it in stimulating the methylation of both cytoplasmic and nuclear proteins including histones (Yang et al., 2020).

In addition to interacting with PRMT5, OXR1 was also shown to interact with PRMT1. Therefore, OXR1 may function to modulate the activity of multiple protein methylases and other regulatory proteins (Finelli et al., 2015; Yang et al., 2020). Thus, the ability of OXR1 to regulate gene expression may result from its protein-protein interactions with PRMT5 potentially remodeling chromatin structure to stimulate gene expression and/or directly stimulate regulatory proteins to carry out their functions. The interaction of OXR1 with PRMT proteins appears to function via the TLDc domain, since OXR1D protein also binds to PRMT5, suggesting this interaction may be shared by all OXR1 isoforms and possibly other TLDc domain proteins.

### ROS Sensing

The function of *OXR1* upstream of a number of regulatory elements led to the suggestion that it may act as a global sensor of oxidative stress in cells (Yang et al., 2015). This hypothesis is intriguing, but evidence for a mechanism of action remains to be determined. The sensor hypothesis implies that *OXR1* must be able to assess some feature of oxidative stress and then signal through its downstream targets that the stress level has become sufficiently severe to trigger the induction of the *OXR1*-dependent response.

OXR1 has two activities that could potentially function as cellular sensors of oxidative stress. One biochemically defined potential sensing mechanism involves a highly conserved cysteine residue, which is Cys^753^ in the mouse *Oxr1* gene. This cysteine is found in the TLDc domain of both OXR1 and NCOA7 and is present in OXR1 of a large number of species including human, mouse, and *Drosophila* (Oliver et al., 2011; Finelli et al., 2016). It is oxidized by H_2_O_2_, but its reactivity is too weak to provide OXR1 with an antioxidant activity sufficiently strong enough to function as an antioxidant protein (Oliver et al., 2011). However, the weak ROS-interacting mechanism of Cys^753^ could function well as a sensing mechanism that leads to activation of OXR1 only when ROS reaches a critical level that is sufficiently strong to oxidize Cys^753^. Several oxidative stress-associated proteins, including DJ-1 and MTK-1, have redox-sensitive cysteine residues that are responsive to ROS levels and which have been shown to modulate the downstream regulatory and catalytic properties of these proteins (Wilson, 2011; Hijioka et al., 2017; Matsushita et al., 2020). Similar biochemical investigations, along with site-directed mutagenesis of Cys^753^ and its potential effects on OXR1-dependent gene expression, have yet to be performed.

The *OXR1* protein can reduce oxidative damage when expressed at high levels in *E. coli* (Murphy and Volkert, 2012) reducing the oxidative damage to DNA that results from low-level, spontaneous ROS production. This suggests a second possible sensing mechanism. The active region required for this activity was localized to a domain within exon 11 that is highly conserved among OXR1 homologs in other species and in the *NCOA7* gene and its homologs. It is unclear how this domain functions at the molecular and biochemical level, but it was demonstrated that this region of OXR1 can reduce the DNA damage resulting from ROS (Murphy and Volkert, 2012), suggesting it may interact directly with ROS, or affect the function of ROS production or detoxification proteins.

It is possible that both regions of OXR1 protein react with ROS directly and function together in cellular sensing. The exon 11 domain may act as an oxidative stress sensing region in the longer isoforms of OXR1. The low reactivity of Cys^753^ raises the possibility that this site may respond only when ROS reach a critical level. It is also the only potential sensing mechanism present in the short forms of OXR1. Determining if these domains function in ROS signaling and OXR1 activation requires further study.

### Subcellular Localization of *OXR1* Protein

Examination of the dynamics of OXR1 localization provides insights into how it might function. Initial localization studies showed that OXR1 is present in the mitochondria of yeast and mammalian cells (Elliott and Volkert, 2004). Mitochondria carry out oxidative phosphorylation for cellular energy production, a process that is the major source of intracellular ROS. Mitochondrial localization of OXR1 led to the suggestion that it may protect cells by its presence in this organelle (Elliott and Volkert, 2004). When an intermediate sized isoform of the human OXR1 protein was tested for its ability to restore peroxide resistance to a yeast *OXR1* deletion mutant, it was able to do so only if it entered the mitochondria. This demonstrates that mitochondrial localization is required in yeast cells for oxidative stress resistance (Elliott and Volkert, 2004). In mammalian cells, the short isoform was shown to localize to the outer membrane of the mitochondria (Wu et al., 2016). Unlike yeast, however, oxidative stress resistance was best achieved in mammalian cells in which *OXR1* was overexpressed and localized to the cytoplasm rather than the mitochondria. It was observed that the cytoplasmic short form can prevent the morphological changes in mitochondria that result from rotenone-induced oxidative stress (Wu et al., 2016).

*OXR1* silencing studies in HeLa cells showed that depletion of *OXR1* by inhibitory RNA (RNAi) caused cellular levels of ROS to rise, which caused mitochondrial DNA damage and initiated a cycle of increased production of ROS by the mitochondria and further cellular damage (Yang et al., 2014). As ROS levels rose, mitochondria became unstable and apoptosis was induced (Yang et al., 2014; Wu et al., 2016). The importance of *OXR1* for mitochondrial stability and ROS control was underscored by the result that *OXR1* depletion by RNAi methods had no effect on peroxide-induced oxidative stress resistance in cells lacking mitochondrial DNA (Yang et al., 2014). The large number of changes resulting from *OXR1* depletion demonstrates that *OXR1* is required for the expression of many genes that protect cells from oxidative stress and the repression of proapoptotic genes (Yang et al., 2014, 2015). Thus, it appears that *OXR1* controls mitochondrial resistance to oxidative stress by regulating oxidative stress responsive genes (Yang et al., 2014).

**FIGURE 2:**
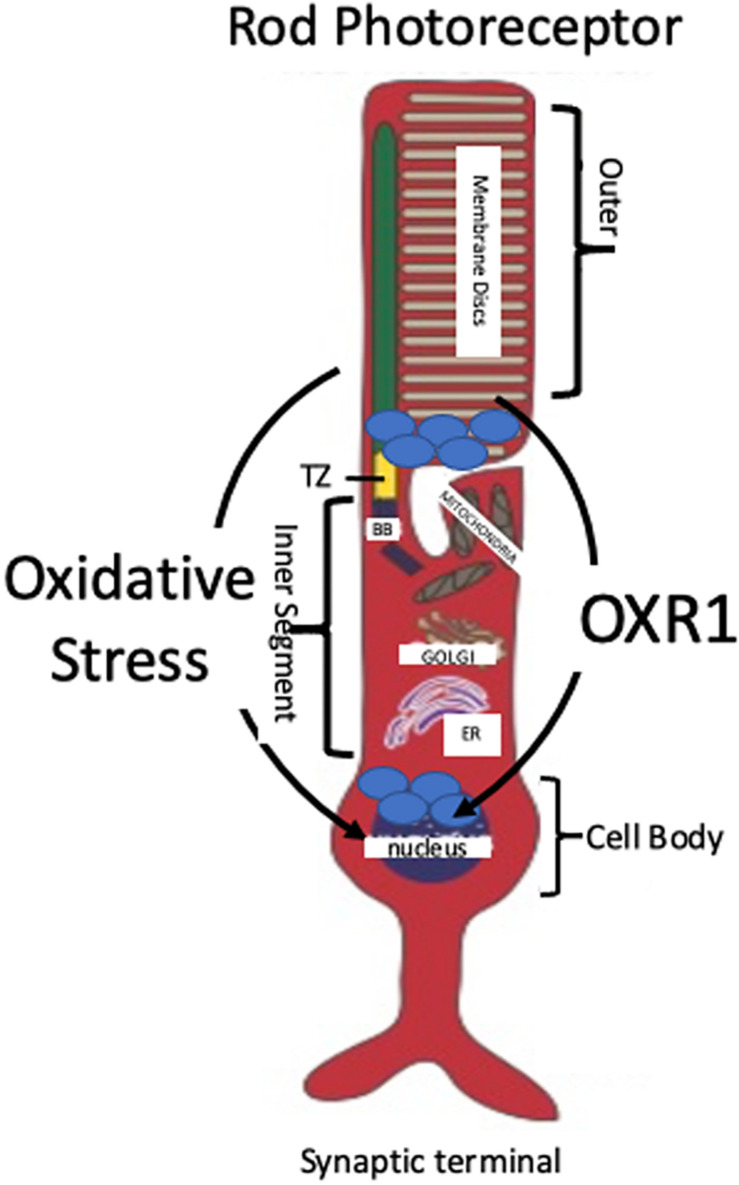
Localization and Migration of OXR1 Upon Oxidative Stress. A typical rod photoreceptor is shown. Rods comprise the majority of photoreceptors in mammals (approximately 98% in mice and 95% in humans). OXR1 is initially located in the inner portion of the outer segment of mouse photoreceptors. Oxidative stress causes OXR1 migration from the outer segment, through transition zone (TZ), into the inner segment and the nucleus (Natoli et al., 2008a). Photo-oxidation of rhodopsin contained within the membrane disks initiates a cascade of chemical reactions that convert light signals into electrical signals transmitted by the photoreceptors to the brain via intermediary neurons. OXR1 localization in the outer segment may serve to monitor ROS levels and trigger a genetic response when ROS level are sufficient to cause stress. Basal body (BB), endoplasmic reticulum (ER).

Localization and migration of OXR1 in response to oxidative stress was examined in the rod photoreceptor cells of C57bl6/J mice, previously described as a model of oxygen-induced retinal degeneration (Natoli et al., 2008a). OXR1 localizes to the inner portion of the outer segments of the photoreceptors prior to the onset of retinal degeneration. Photoreceptor outer segments lack mitochondria indicating a cytoplasmic localization of OXR1 in non-stressed cells (Natoli et al., 2008a; Figure 2). The outer segments contain the photoactive pigments needed for light perception in layers of disks. Extensive photo-oxidation reactions occur in the outer segments and ROS levels are high, especially when the eye is exposed to light of shorter wave lengths (Roehlecke et al., 2013). All protein synthesis occurs in the inner segment of the photoreceptor cell. A transition zone links the inner and outer segments and all cellular components synthesized in the inner segment that are required for outer segment’s visual functions must be transported through the transition zone. As described previously, incubation of C57bl6/J mice in high oxygen triggers neurodegeneration of the photoreceptor cells. As their degeneration progresses, *OXR1* protein migrates from the outer segment back into the inner segment, passing through the transition zone and entering the nucleus (Natoli et al., 2008a; Figure 2). Nuclear localization is consistent with the known regulatory effects of *OXR1* on gene expression during oxidative stress (Yang et al., 2014, 2015). While localization to outer segments is unique to photoreceptor cells, its presence in the nucleus of HeLa cells was described by Yang et al. (2020). The localization of OXR1 in the outer segments of non-stressed photoreceptor cells may be associated with the high levels of ROS present and the photo-oxidation that occurs in this region of the cell. It is not known which isoform(s) of OXR1 showed this localization pattern because the antibody used in this study recognized the TLDc domain and therefore detects all major isoforms of *OXR1*.

OXR1 localizes to two highly oxidizing cellular compartments, the outer segments of photoreceptor cells and the mitochondria of other cells (Elliott and Volkert, 2004; Natoli et al., 2008a; Yang et al., 2014; Wu et al., 2016). OXR1 also is found in the cytoplasm, however, its function in this compartment still requires normal mitochondrial activity, since cells lacking mitochondrial DNA, which inhibits mitochondrial function, are not sensitized to peroxide treatment upon depletion of OXR1 (Yang et al., 2014). This suggests OXR1 is monitoring mitochondrial oxidative stress while present in the cytoplasm, possibly responding to a signal released from mitochondria upon stress.

Combining these studies suggests that OXR1 functions to monitor oxidative stress levels in highly oxidizing compartments of cells. in photoreceptor cells it is monitoring the highly oxidizing environment of the outer segment. In other cell types, OXR1 is monitoring oxidative stress levels, either by its presence in mitochondria, or by monitoring release of mitochondrial oxidative stress signals into the cytoplasm. Moreover, the mitochondrial localized short forms may monitor oxidative stress within the mitochondria, while the cytoplasmic long forms monitor mitochondrial stress levels via signals released into the cytoplasm.

### Direct Protein-Protein Interactions Contribute to the Role of OXR1 in Neurodegeneration

A third molecular function for *OXR1* has been demonstrated in a series of papers from the lab of Peter Oliver (Finelli et al., 2019; Svistunova et al., 2019; Williamson et al., 2019). This group has shown that *OXR1* interacts directly with a number of proteins including proteins involved in ROS production and detoxification. OXR1 also appears to have additional protein-protein interactions that may contribute to the delay of disease onset and death in the ALS mouse model carrying *TDP-43* mutations. Finelli et al. (2015) first showed that OXR1D (Oxr1C in mouse) binds to wild type TDP-43 and FUS proteins, two ALS associated proteins. TDP-43 is a protein involved in multiple mRNA production and processing steps (Buratti and Baralle, 2008). The mutant *TDP-43*^*M337V*^ protein is associated with ALS and accumulates in cytoplasmic stress granules. Raising the levels of OXR1 protein in cells over expressing the *TDP-43^M337V^* allele increases localization of TDP-43^M337V^ protein in the nucleus and decreases localization of TDP-43^M337V^ in stress granules. The M337V mutation of *TDP-43* results in aberrant splicing of the RNA of the *Mtfr-1* (mitochondrial fission receptor-1) gene. To determine if increasing OXR1 levels and improving nuclear localization of TDP-43 restored normal TDP-43 function, *Mtrf-1* splicing was examined. The RNA of this gene was again spliced normally indicating that increasing OXR1 protein levels restores TDP-43’s nuclear localization and its splicing function (Finelli et al., 2015; Williamson et al., 2019).

Next, Williamson et al. (2019) tested the ability of OXR1 over expression to alleviate the symptoms of ALS in a mouse that carries the human M337V mutant *TDP-43* as a transgene. As is the case in cell lines with this mutation, the mutant human TDP-43^M337V^ protein localizes to stress granules in primary neurons from this mouse. Crossing this *TDP-43^*M*337V^* mouse with a mouse that over expresses Oxr1 in neurons yielded primary neurons with increased TDP-43^M337V^ localization in the neuronal nuclei. The *TDP-43^*MV*337V^* mouse that over expresses OXR1 from the transgene had decreased muscle denervation, decreased neuromuscular degeneration, decreased neuroinflammation and improved motor function. At present it is unclear if the reduction in ROS levels seen when OXR1 levels were increased (Finelli et al., 2016), the improved localization of TDP-43^m337V^ seen in cells (Finelli et al., 2015), or possibly both of these consequences of *OXR1* overexpression contribute to the suppression of the phenotype of the TDP-43^M337V^ mouse.

### Protein-Protein Interactions May Modulate ROS Levels

The Oliver lab has also provided compelling evidence that *OXR1* regulates the function of the key metabolic enzyme glucose-6-phosphate isomerase (GPI) through direct protein-protein interactions (Finelli et al., 2019). The human brain consumes large amounts of glucose through oxidative catabolism and disruption of normal glucose metabolism is a feature of several neurodegenerative diseases (Mergenthaler et al., 2013). OXR1 deletion mutant mice showed significant neurodegeneration that was preceded by an inability to down regulate glycolytic activity under oxidative stress. They also showed a disruption in the levels of many metabolites produced downstream of glucose-6-phosphate during its metabolism. Glucose-6-phosphate is an early intermediate in glycolysis and the pentose phosphate pathway, two pathways associated with ROS production. Both pathways are impacted by the activity of GPI, a key enzyme in the early stages of glucose metabolism. The investigators demonstrated that OXR1 physically interacts with GPI through its TLDc domain and that this interaction modulates the neuroprotective and cytokine activities of GPI through its level of oligomerization. *OXR1* binding to GPI reduces multimeric forms associated with GPI’s glycolytic activity in favor of monomeric forms responsible for GPI’s cytokine activity, a shift that was shown to decrease in *OXR1* mutants.

Like GPI, peroxiredoxin 2 (Prdx2) is a multifunctional protein. It has a powerful antioxidant activity and the ability to act as a molecular chaperone. These activities, along with its high level of expression in neurons, make it an important target for studies of neurodegeneration, which is consistently associated with aberrant levels of oxidative stress and protein aggregation. Svistunova et al. (2019) showed that OXR1 binds to Prdx2 and modulates its activities. This study provided evidence that OXR1 acts as a functional switch for Prdx2 in the cerebellum, fine-tuning its activity by modulating both its degree of oligomerization and two key post-translational modifications, S-nitrosylation and overoxidation. In *OXR1* knockout mice, S-nitrosylation of Prdx2 was high while overoxidation was low, a combination that inhibits Prdx2-associated antioxidant activity and may result in high ROS in cells and subsequent neurodegeneration. As expected, increased expression of *OXR1* reversed these modifications.

### *OXR1* and the Immune Response

Liu et al. (2015) found that, in addition to affecting the expression of oxidative stress resistance genes, the elevated immune response is rescued by Oxr1 over expression in mouse models of ALS. They find that neuronal over expression of *Oxr1* in the SOD1^G93A^ mutant reduced expression of several markers of macrophage activation and microgliosis that were induced in *SOD1^*G*93A^* mouse. STAT3 phosphorylation and its subsequent activation of downstream immune responses occurs in the SOD1^G93A^ mice but are reduced when *Oxr1* is over expressed in neurons. Controlling neuroinflammation and the immune response can further contribute to increased viability of neurons. It remains to be determined if this is a direct regulatory effect of *OXR1* expression or if *OXR1*-mediated repression of oxidative stress in neurons reduces the need for activation of an immune response (Staal et al., 1994; Reuter et al., 2010).

### Hypothetical Model of the *OXR1* Regulatory Cascade

**FIGURE 3:**
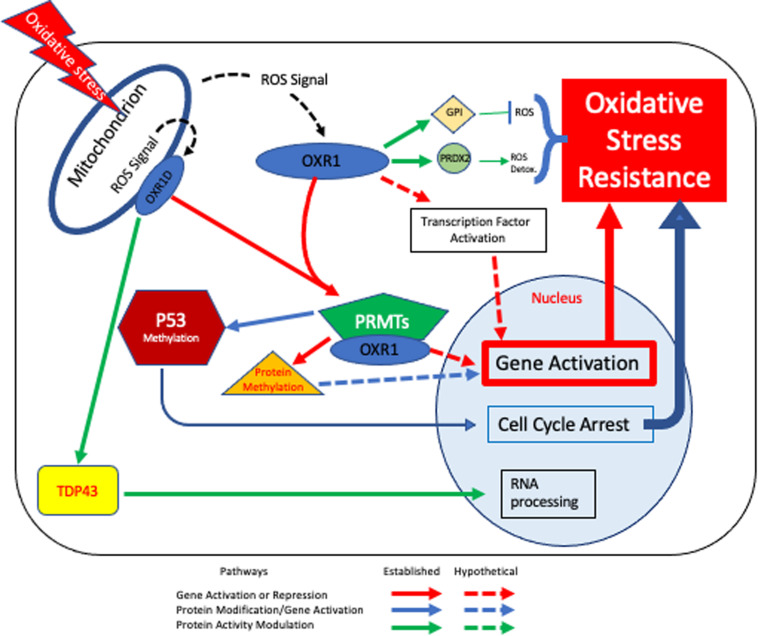
OXR1-mediated Oxidative Stress Resistance Model. Based on our current understanding, OXR1 functions in multiple pathways that contribute to oxidative stress resistance. In this model cells experience oxidative stress, either due to external or internal factors affecting ROS levels and respond in an OXR1-dependent fashion. Mitochondria are required for OXR1 function (Yang et al., 2014) (upper left). When oxidative stress occurs, mitochondrial bound and/or cytoplasmic OXR1 forms act as sensors that respond to the signal (possibly ROS) and triggers downstream protective pathways. Pathways shown with solid lines are supported by multiple lines of evidence. Dashed lines represent hypothetical pathways. The red arrows indicate pathways involved in activation or repression of gene expression. One set of red arrows illustrate direct protein-protein interactions of OXR1 with transcription factors to alter gene expression. A second red pathway indicates regulation by OXR1 interaction with PRMT5, PRMT1 and possibly other PRMT proteins. PRMT proteins methylate histones and modulate chromatin structure and proteins other than histones, possibly including transcription factors. Blue arrows depict the PRMT5 activation of p53 to arrest the cell cycle, which is necessary for efficient DNA repair and regulation of apoptosis (Yang et al., 2020). Green arrows depict interactions of OXR1 with other proteins to modulate their activities (Finelli et al., 2015, 2019; Svistunova et al., 2019; Williamson et al., 2019).

Combining the various known roles of OXR1 in regulating cellular responses to oxidative stress leads to a potential model of how OXR1 may function in neurons, which is presented in Figure 3. If the proposal that OXR1 is a sensor of oxidative stress is correct (Yang et al., 2015), OXR1 proteins may sense the levels of ROS in locations like the photoreceptor outer segments and mitochondria (Elliott and Volkert, 2004; Yang et al., 2014; Wu et al., 2016), possibly by the interaction of ROS with the exon 11 domain (Murphy and Volkert, 2012), by direct oxidation of Cys^753^ (Oliver et al., 2011), or both. In Figure 3 we include both the possibility that mitochondrially-associated OXR1D directly monitors mitochondrial stress and that cytoplasmic OXR1 proteins may respond to signals released by mitochondria as they become stressed. The possibility that the signal sensed by OXR1 may be ROS, or another product or products resulting from oxidative stress or ROS activity remains to be determined.

Regardless of whether OXR1 acts directly as a sensor of ROS or responds to an unknown product of ROS activity or oxidative stress, it is clear that a stress response is stimulated by OXR1 and that this results in prevention cell death by altering gene expression and through direct protein-protein interactions. OXR1 has been shown to interact with PRMT5 and possibly PRMT1. These interactions may modulate transcription of genes by altering chromatin structure (Yang et al., 2020). This may be a key mechanism by which OXR1 acts to stimulate transcription of oxidative stress resistance gene expression. There are, however, several additional mechanisms by which OXR1 may control gene expression. Since PRMT proteins also modify proteins other than histones (Lorton and Shechter, 2019), the OXR1-PRMT interactions could stimulate gene expression by activating regulatory proteins via methylation, as has been demonstrated for p53 (Yang et al., 2020). OXR1 also binds directly to a number of proteins besides the PRMTs, including several transcriptional regulatory proteins (Finelli et al., 2015; Yang et al., 2020). Therefore, it could also act independently of PRMTs to directly stimulate regulatory proteins that control expression of oxidative stress resistance genes. Both functions are possible and OXR1 likely acts through PRMT-dependent and PRMT-independent gene regulatory mechanisms. Its close homolog NCOA7 functions as a nuclear coactivator that binds to the estrogen receptor regulatory complex, which then binds to and activates the promoters of specific genes (Shao et al., 2002). A direct transcriptional coactivation role for OXR1 has not been tested and remains a possibility.

Additionally, OXR1’s protein-protein interactions modulate the activities of ROS production and detoxification proteins, which may affect ROS levels, as well as other proteins involved in neurodegeneration (Finelli et al., 2015, 2019; Svistunova et al., 2019; Williamson et al., 2019; Yang et al., 2020). Thus, OXR1 provides a direct protection from neurodegenerative diseases in addition to its role in regulating gene expression. While the consequences of OXR1’s interactions with TDP-43, peroxiredoxin, and GPI proteins have been characterized and shown to be protective (Finelli et al., 2015, 2019; Svistunova et al., 2019; Williamson et al., 2019), the consequences of its interaction with a number of other proteins are less well understood. The underlying theme does, however, emerge that OXR1 is intimately tied to controlling the cell’s response to oxidative stress and neurodegeneration at several different levels. It causes changes in gene expression that increase oxidative stress resistance and promotes protein activities that protect cells from oxidative stress and neurodegeneration.

The role of OXR1 as a key player in oxidative stress resistance and neurodegeneration is becoming clear, however much remains to be learned about how oxidative stress is sensed in the cell, what the sensing signal is, and how OXR1 functions to alter gene expression to confer resistance and tolerance of oxidative stress. OXR1 also appears to interact with other proteins that are not directly related to oxidative stress but do play roles in neurodegenerative diseases. Figure 3 combines the various known and hypothetical functions of OXR1 into a cohesive picture that includes its possible role as a sensor, its interaction with various proteins, and the known and hypothetical consequences of the various protein-protein interactions described above. Current efforts to examine the various mechanisms of action are underway and should greatly aid our understanding of OXR1 functions.

## Therapeutic Potential of *OXR1*

### *OXR1* Gene Therapy

Attempts to treat neurodegenerative diseases with antioxidants have largely failed (Group, 1993; Lloret et al., 2009). One possible explanation is that ROS are involved in many metabolic pathways and functions as signaling molecules (D’Autreaux and Toledano, 2007). Treatment with antioxidants is not selective and may interfere with oxidative metabolism and cellular signaling pathways that employ ROS in addition to detoxifying deleterious ROS. Controlling ROS levels by activating cellular oxidative stress resistance mechanisms may be more selective, allowing normal oxidative metabolism and ROS signaling to proceed while detoxifying and reducing levels of harmful ROS.

*OXR1* gene therapy could treat multiple neurodegenerative diseases, because oxidative stress is a common event that leads to neuronal cell death (Sayre et al., 2008; van Horssen et al., 2011; Punzo et al., 2012; Calderon et al., 2017; Liu et al., 2017; Puspita et al., 2017; Angelova and Abramov, 2018; Lupoli et al., 2018; Wu et al., 2018). Increasing OXR1 expression to higher levels by introducing extra copies using an adeno-associated viral (AAV) vector that expresses a microRNA resistant form of *OXR1* could potentially be used to treat such diseases. This can be beneficial, in cells that have repressed their normal OXR1 levels by a miRNA or through protein destabilization. Since protection from degeneration also occurs upon elevating OXR1 protein levels further in cells where its expression is already high, OXR1 gene therapy can potentially also be beneficial in cells where it is not repressed (Finelli et al., 2015; Liu et al., 2015; Williamson et al., 2019). Moreover, high level expression of OXR1 in brain neurons was not associated with any detectable pathological consequences, suggesting elevating OXR1 levels is safe (Oliver et al., 2011; Liu et al., 2015).

AAV-*Nrf2* gene therapy has been tested in a mouse model of retinal degeneration as a potential approach to treat multiple retinal degenerative diseases by treating oxidative stress (Xiong et al., 2015). Like *OXR1, Nrf2* is a regulatory gene that controls genes required for oxidative stress resistance. It has been used to treat retinal degeneration in *rd1* and *rd10* mutant mice. These mutations cause loss of vision within approximately 4 and 8 weeks, respectively. Introduction of *Nrf2* into the retina of these mice resulted in a retention of some visual activity in the *rd10* mouse, as measured by electroretinography. Two oxidative stress resistance genes regulated by *Nrf2* are the *GPX2* and *HMOX1* genes. OXR1 is also required for the expression of these genes and controls a wide array of additional genes that contribute to resistance and viability.

Many current gene therapy approaches focus on identification of a defective gene and introduction of a wild type copy to restore function. Such therapies have a limited application. For example, gene therapy for Leber’s congenital amaurosis (LCA), a retinal degenerative disease, recently received FDA approval. This treatment introduces a wild type copy of the *RPE65* gene (Cideciyan, 2010; Boye et al., 2015; Feuer et al., 2016; Weleber et al., 2016). While this is the most common gene mutation that causes LCA, there are at least 17 other mutant genes known to cause this disease that this therapy cannot treat (Cideciyan, 2010; Kumaran et al., 2017). Retinitis pigmentosa poses an even bigger problem as over 50 different genes have been identified that cause this retinal degenerative disease (Petit et al., 2016).

While gene therapy for retinal degenerative diseases is currently achievable, the use of such therapies in the brain will require the development of vectors capable of crossing the blood-brain barrier, or the use of more invasive methods, such as direct injection. Should it be possible to safely overcome these issues, OXR1 gene therapy could treat multiple diseases in which oxidative stress contributes to neurodegeneration.

In addition to adding extra copies of *OXR1* using an AAV vector, it is also possible to increase *OXR1* gene expression by other genetic and biochemical means. Genetic approaches, such as that used by Mo et al. (2019) and described above, to block the action of inhibitory microRNAs by introducing an antagomir, can restore *OXR1* expression in diseases where microRNAs play a role. In such diseases it is potentially possible to use CRISPR methods to alter the sequences of the *OXR1* gene that encode the microRNA targets of *OXR1* mRNAs. CRISPR has recently been used to increase transcription by altering the promoter activity of genes (Liao et al., 2017; Moreno et al., 2018; Bohm et al., 2020). Since increasing *OXR1* expression is the goal of all of these therapies, using CRISPR methods to genetically alter the promoter function to increase transcription, or searching for small molecules or other drugs that increase OXR1 protein levels or stability are also possible strategies worthy of exploration.

## Conclusion

The experiments that have been done in *OXR1-*deletion mutant mice, ALS mouse models, the MPTP-treated Parkinson’s mice, the ischemic rat model, and in mouse models of retinal degenerative diseases underscore the important role that *OXR1* plays in protecting neuronal cells from degeneration in multiple diseases. It also demonstrates that neuronal protection results from its role in oxidative stress resistance as well as its protein-protein interactions and effects on neuroinflammation. These studies strongly suggest that *OXR1*-mediated gene therapy can protect neurons from degeneration in such diseases. The Parkinson’s and ALS mouse models further demonstrate that increasing *OXR1* expression alleviates physical symptoms associated with neurodegeneration (Liu et al., 2015; Li et al., 2017; Jiang et al., 2019; Williamson et al., 2019). These studies clearly make *OXR1* an appealing therapeutic target for the treatment of neurodegenerative diseases. Further studies of the molecular and cellular mechanisms of OXR1 are needed to better understand how it confers oxidative stress resistance to cells. Such studies can reveal additional potential points of therapeutic intervention to modulate the expression of oxidative stress resistance genes for the treatment of neurodegenerative diseases. Current genetic methods make such therapies a realistic possibility in the near future.

## Author Contributions

MV and DC contributed to the article, developed the model, and approved the submitted version.

## Conflict of Interest

The authors declare that the research was conducted in the absence of any commercial or financial relationships that could be construed as a potential conflict of interest.

## Footnotes

1 https://www.proteinatlas.org
